# Acupuncture to treat asymptomatic hyperuricemia: A protocol for systematic review and meta-analysis of randomized controlled trials

**DOI:** 10.1097/MD.0000000000024719

**Published:** 2021-02-12

**Authors:** Ji Hye Hwang, Kwang Ho Lee, Dong Woo Nam, Ho Sueb Song

**Affiliations:** a Department of Acupuncture & Moxibustion Medicine, College of Korean Medicine, Gachon University, Seongnam, Republic of Korea; b Department of Acupuncture & Moxibustion Medicine, College of Korean Medicine, Sangji University; c Department of Acupuncture & Moxibustion Medicine, College of Korean Medicine, Kyung Hee University.

**Keywords:** acupuncture, gout, hyperuricemia, protocol, systematic review

## Abstract

**Background:**

Hyperuricemia (HUA) plays an important role in metabolic syndrome, cardiovascular disease, and kidney disease. HUA without resulting gout is referred to as asymptomatic HUA. The purpose of the present systematic review protocol is to provide methods to assess the effectiveness and safety of acupuncture-based treatment for asymptomatic HUA.

**Methods:**

To identify randomized controlled trials (RCTs) involving acupuncture-based treatment for asymptomatic HUA, a search will be carried out using the following eight electronic databases: MEDLINE, EMBASE, Cochrane Library, Korea Med, Oriental Medicine Advanced Searching Integrated System, Korean Studies Information Service System, China National Knowledge Infrastructure, and Japanese Institutional Repositories Online. Manual search and email contact with the author will also be conducted if necessary. Studies will be selected based on predefined criteria and summarized data regarding study participants, interventions, control groups, outcome measures, side effects, and risk of bias. No language restrictions will be imposed. Studies that evaluated any type of acupuncture will be eligible for inclusion, and the primary outcome will be the blood uric acid level. The methodological quality of the included RCTs will be assessed using the Cochrane risk of bias tool.

**Results:**

The present study will evaluate the efficacy and safety of acupuncture to treat HUA.

**Conclusion:**

Our findings will establish the evidence for acupuncture-based treatment of HUA and will be informative for patients with HUA, clinicians, policy makers, and researchers.

**Registration number:**

reviewregistry1054.

## 1 Introduction

Serum uric acid levels depend on the balance between synthesis and excretion of uric acid, which is the final product of purine metabolism.^[1]^ Hyperuricemia (HUA) is characterized by abnormally elevated serum uric acid levels^[1]^ and is a leading cause of gout,^[2]^ a progressive metabolic disease characterized by symptomatic HUA and deposition of monosodium urate crystals in the joints and soft tissues. Gout is caused by an imbalance in the absorption, synthesis, or excretion of uric acid.^[3]^ It has a prevalence of 3.2% in the UK and Spain, 4.7% in Greece, 3% in Canada, 3.9% in the United States, 1.4% in Australia, 6% in New Zealand, and 4.92% in Taiwan.^[4]^ It causes deformity and disability in the joints, as well as complications such as kidney diseases and urolithiasis.^[5]^

HUA is defined in Korea^[5]^ and China^[6,7]^ as a serum uric acid level of ≥7 mg/dl for men and of ≥6 mg/dl for women. HUA that has not led to gout is referred to as asymptomatic HUA.^[6,7]^ The prevalence of HUA was 21.6% among men and 8.6% among women in China, according to a 2011 meta-analysis.^[8]^ Similarly, a 2010 study showed a prevalence of 12.7% in the United States.^[9]^ In a 2017 study conducted on health screening attendees at tertiary medical institutions in Korea, the prevalence of HUA was 14.3% in men and 2.2% in women. The same survey stated that 16.6% of men and 6.7% of women reported gout.^[5]^ More recently, HUA has been associated with metabolic syndrome, cardiovascular disease, high blood pressure, kidney disease, and stroke,^[2,10–13]^ and the many potential complications of HUA can significantly increase medical costs.^[5,14]^ Therefore, it is crucial that clinicians gain a better understanding of HUA.

Because of its established association with HUA, gout, and decrease in SU in its chronic management, urate-lowering therapy is a prime pillar of chronic gout management.^[15]^ Currently, *long*-*term* HUA *treatment* is aimed at regulating the *activity* of *key enzymes involved* in the *metabolism* and *excretion* of *uric acid, such as* xanthine oxidase and urate-anion exchanger.^[16,17]^ However, the side effects of these treatments include liver function abnormalities, diarrhea, headache, nausea, and rash,^[17,18]^ so many investigators now conduct research into new treatment strategies with fewer side effects.^[17]^

Complementary and alternative medicine (CAM) has become more widely advocated because there is growing demand for non-pharmacological approaches. Acupuncture is based on the theories of traditional Korean medicine (TKM) and traditional Chinese medicine (TCM). It is a mainstream CAM therapy and is commonly used to treat gout in Asian cultures.^[19]^ In TKM and TCM, HUA belongs to the arthromyodynia or “Bi” disease category. For thousands of years, acupuncture has been used to treat a variety of clinical conditions, including arthromyodynia or “Bi” diseases, based on TCM and TKM theories.^[20]^ In addition, clinical and experimental studies have reported that acupuncture has therapeutic effects on metabolic syndrome,^[21]^ cardiovascular diseases such as arrhythmias,^[22]^ heart failure,^[23]^ hypertension,^[24]^ chronic kidney disease,^[25]^ and pain control in acute gouty arthritis.^[26,27]^

A systematic review and analysis of asymptomatic HUA has been carried out in the field of herbal medicine.^[14]^ However, although acupuncture has been used to treat asymptomatic HUA,^[28]^ there is currently insufficient evidence to systematically review the efficacy and safety of such treatment. Therefore, the proposed review will focus on evaluating the efficacy and safety of various acupuncture therapies in the treatment of asymptomatic HUA.

## 2 Methods

### 2.1 Study registration

We will conduct this systematic review report in accordance with the Preferred Reporting Items for Systematic Reviews and Meta-Analyses protocols (PRISMA-P).^[29]^ The protocol was registered in the Research Registry (registration number: reviewregistry1054).

(https://www.researchregistry.com/browse-the-registry#registryofsystematicreviewsmeta-analyses/

### 2.2 Ethics

As the study will review published literature, no ethical approval is required, as there will be no patient recruitment and no personal data collection.

### 2.3 Eligibility criteria

#### 2.3.1 Types of participants

Adult patients (aged ≥18 years) diagnosed with asymptomatic HUA will be included, with no restrictions on any other conditions, such as sex, country of origin, education status, or severity of symptoms. Patients will be excluded if they have overweight or obesity, hypertension, diabetes, dyslipidemia, history of gout or HUA requiring medication, or chronic kidney disease.

#### 2.3.2 Types of interventions and controls

Studies that evaluate any type of acupuncture will be eligible for inclusion, including acupuncture, electro-acupuncture, auricular acupuncture, pharmacopuncture, acupotomy, bee venom acupuncture, blood-letting, cupping, moxibustion, fire needling, and warm acupuncture. RCTs that include acupuncture-based treatment alone or as an adjunct to other treatments will be included if they provided the same treatment to the control and intervention groups. Trials comparing acupuncture-based treatment with any type of control intervention will also be included.

Trials with positive comparators and placebo control groups will be included. Trials comparing acupuncture-based treatment with any type of control intervention will also be included. Other interventions will include herbal medicine, chuna, diet therapy, and physical therapy, as well as conventional treatments such as uric acid-lowering therapy and xanthine oxidase inhibitors (e.g., allopurinol).

#### 2.3.3 Types of studies

Prospective RCTs that evaluate the effectiveness of acupuncture-based treatment for asymptomatic HUA will be considered, including acupuncture, electroacupuncture, auricular acupuncture, pharmacopuncture, acupotomy, bee venom acupuncture, blood-letting, cupping, moxibustion, fire needling, and warm acupuncture. Case reports, observational studies, cross-sectional studies, pilot studies, and systematic review protocols will be excluded.

#### 2.3.4 Outcomes and prioritization

The primary outcome will be blood uric acid level, while the following additional outcomes will also be recorded:

1. serum/urine creatinine
2. albumin-to-creatinine ratio
3. blood urea nitrogen
4. serum lipid profiles, including total cholesterol (TC), triglyceride (TG), high-density lipoprotein cholesterol (HDL-C), and low-density lipoprotein cholesterol (LDL-C)
5. fasting plasma glucose (FPG)
6. HbA1c
7. xanthine oxidase
8. urate-anion exchanger (URAT-1)
9. quality of life
10. adverse events

### 2.4 Data sources and search strategy

**Figure 1:**
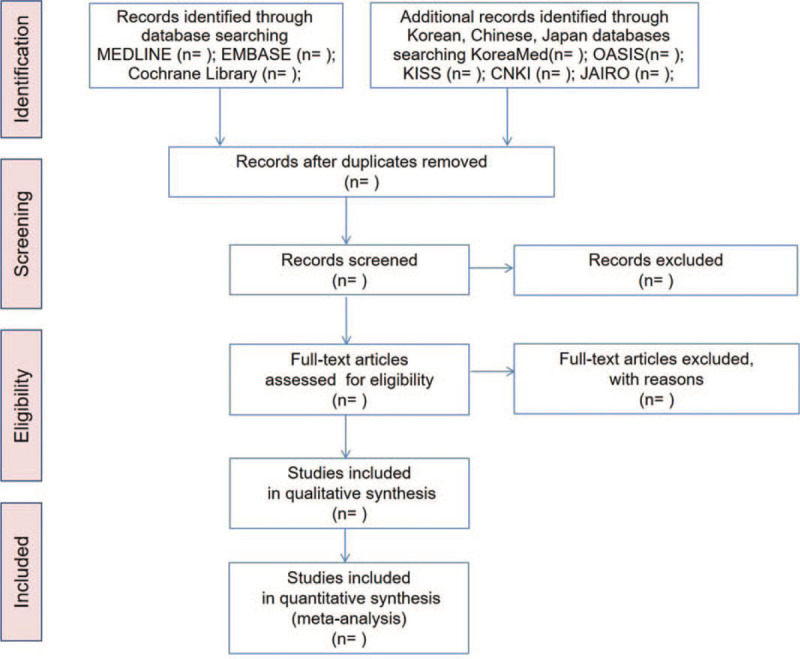
PRISMA flow diagram. CNKI = China National Knowledge Infrastructure, JAIRO = Japanese Institutional Repositories Online, KISS = Korean Studies Information Service System, OASIS = Oriental Medicine Advanced Searching Integrated System.

The databases and search terms will be determined through discussion between the authors before the literature searches are executed. Two independent researchers will perform electronic literature searches, study selection, data extraction, and quality assessment. The following electronic databases will be searched for studies, from inception to the present date: MEDLINE, EMBASE, Cochrane Library, 3 Korean databases, Korea Med, Oriental Medicine Advanced Searching Integrated System, Korean Studies Information Service System, China National Knowledge Infrastructure, and Japanese Institutional Repositories Online. Prospective RCTs that evaluated the effectiveness of acupuncture-based treatment for asymptomatic HUA will be included in this review. No language restrictions will be imposed (Fig. 1).

### 2.5 Data extraction

We will review all searched articles to evaluate their eligibility for inclusion. In the case of uncertainties, the authors will be contacted for further information. After the selection of studies, we will extract the following data from the selected articles: author, year of publication, study design, participants (age, gender), diseases or disorders, intervention, control intervention, outcome measures, main results, and adverse events.

Two independent researchers will extract the data using a recognized data extraction form that will be agreed on by all reviewers. This form will include the author name (s), patient age, country, year of publication, participant characteristics, intervention, method of randomization, blinding, control treatment, main outcomes, and adverse events. The reviewers will perform a quality assessment using a predefined data extraction form.

### 2.6 Data synthesis and analysis

Differences between the intervention and control groups will be assessed. Mean differences (MDs) with 95% confidence intervals (CIs) will be used to measure the effect of treatment in cases of continuous data. Other forms of data will be converted into MDs. To analyze outcome variables on different scales, we will use standard MDs with 95% CIs. For dichotomous data, we will present treatment effects as relative risks (RRs) with 95% CIs; other binary data will be converted into RR values.

All statistical analyses will be conducted using Cochrane Collaboration's software program Review Manager version 5.3 (Copenhagen, The Nordic Cochrane Center, the Cochrane Collaboration, 2014) for Windows. We will contact the corresponding authors of studies that have missing information to acquire and verify the data whenever possible. When appropriate, we will pool the data across studies to conduct a meta-analysis using fixed or random effects. We will use GRADE Pro software from Cochrane Systematic Reviews to create a Summary of Findings table.

Intention-to-treat analyses, which includes all randomized patients, will be carried out to address missing data. A last observation carry-forward analysis will be applied in cases with missing outcome data. The original source or published trial reports of the data will be reviewed in cases where individual patient data are unavailable initially.

### 2.7 Assessment of risk of bias in individual studies

Risk of bias will be assessed using the Cochrane risk of bias assessment tool, which takes into account random sequence generation, allocation concealment, blinding of participants and personnel, blinding of outcome assessment, incomplete outcome data, selective reporting, and other sources of bias. The results of the assessments will be presented using scores of “L” indicating a low risk of bias, “U” indicating an uncertain risk of bias, and “H” indicating a high risk of bias. Any disagreement will be resolved through discussion among all authors; if any disagreement regarding selection cannot be resolved through the discussion, an arbiter will make the final decision.

### 2.8 Analysis of subgroups or subsets

If considerable heterogeneity is identified (*P* < .1 via χ^2^ test or Higgins *I*^2^ ≥ 50%), subgroup analyses will be performed.

## 3 Discussion

Over the past 20 years, the prevalence of gout and HUA has increased in developed countries,^[3]^ and HUA plays an important role in metabolic syndrome, cardiovascular disease, and kidney disease.^[2,10–13]^ It is crucial that researchers and clinicians better understand HUA, as the disease can lead to loss of work, increased medical costs, and diminished quality of life.

Conventional treatments for HUA have several side effects and limitations, so more attention should be paid to CAM. In this regard, acupuncture of various types has been used to treat HUA for a long time, but evidence such as systematic review is lacking.

Therefore, in the present systematic review and meta-analysis, we intend to evaluate the efficacy and safety of acupuncture in the treatment of HUA by analyzing clinical symptoms and laboratory indicators. We hope that our findings will be useful as a resource to guide optimized clinical treatment strategy for health policy makers, clinical practitioners, patients, and researchers. In future, patients with HUA could be managed using appropriate acupuncture prescribed by physicians based on good evidence. We expect that the study will be used to establish an integrated model combining both Eastern and Western treatment of HUA.

## Author contributions

HSS conceived the study and developed the criteria, JHH, DWN, and KHL searched the literature, and analyzed the data. JHH wrote the protocol, and JHH and HSS revised the manuscript. All authors have read and approved the final manuscript.

**Conceptualization:** Ho Seub Song.

**Investigation:** Jihye Hwang.

**Methodology:** Dong Woo Nam, Kwang Ho Lee.

**Resources:** Dong Woo Nam, Kwang Ho Lee.

**Supervision:** Ho Seub Song.

**Writing – original draft:** Jihye Hwang.

**Writing – review & editing:** Jihye Hwang, Ho Seub Song.

## Abbreviations

Abbreviations: CAM = Complementary and Alternative Medicine, CI = confidence interval, GRADE = Grading of Recommendations Assessment, Development and Evaluation, HUA = hyperuricemia, MD = mean difference, PRISMA = Preferred Reporting Items for Systematic Reviews and Meta-Analysis, RCT = randomized controlled trial, RR = relative risk, TCM = Traditional Chinese Medicine, TKM = Traditional Korean Medicine.
